# Female, juvenile, and calf sperm whale *Physeter macrocephalus* (Linnaeus 1758) records from Ireland

**DOI:** 10.1002/ece3.70056

**Published:** 2024-09-01

**Authors:** Seán A. O'Callaghan, Bogna Griffin, Stephanie Levesque, Martin Gammell, Joanne O'Brien

**Affiliations:** ^1^ Marine and Freshwater Research Centre Atlantic Technological University Galway City Ireland; ^2^ Marine Institute Oranmore Galway Ireland; ^3^ Irish Whale and Dolphin Group Kilrush Clare Ireland

**Keywords:** sperm whale, calf, juvenile, female, Ireland, sighting, stranding, whaling

## Abstract

Sperm whales spatially segregate by sex and social behavior as they mature. In the North Atlantic, male whales move to higher latitudes as far as Svalbard at 80° N, while females and young whales typically remain around lower latitudes below 40–45° N. The Azores, Madeira, and the Canary Islands constitute important nursery grounds for female and young sperm whales. Irish waters represent a midpoint for this species’ spatial segregation in the Northeast Atlantic, where the species occurs along the submarine canyon systems to the west of the country. Historically, just male whales were thought to be found in this region between 51 and 55° N, but one adult female was caught by commercial whalers in 1910, and a 5.49 m calf was found stranded in 1916. Between 1995 and 2023, 10 female sperm whales have been stranded around the coast of Ireland. Eight of these whales have been stranded since 2013, and there has been at least one stranding per year between 2019 and 2023. Four of these strandings have occurred in Donegal in the northwest of Ireland, indicating the presence of female whales along the continental shelf off this region. Two females were stranded within a day of each other and were found in similar states of decomposition in February 2022, indicating that they may have been part of the same group rather than being lone vagrant individuals. Sperm whale calves and juveniles were also sighted in Irish waters in 2001, 2004, and 2010 in the Rockall Trough, along the Porcupine Bank and Goban Spur, where between 1 and 3 individuals were observed on four occasions while one calf live stranded in 2004. These records indicate a historical presence of female and young sperm whales in this region but that an apparent increase in occurrence has taken place over the past decade.

## Graphical abstract

Ireland has historically been viewed as a region where only male sperm whales were present. There have however been historical records of female and calf sperm whales from 1910 and 1916. More recently, there has been a trend of female strandings in Ireland since 1995 where 7 of the 10 events have taken place since 2013 indicating a shift in the sperm whale population structure off Ireland.

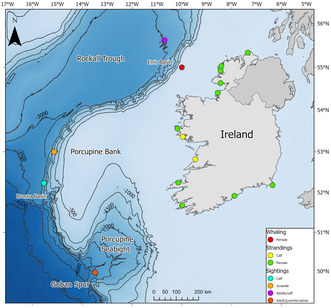

## 1 INTRODUCTION

The sperm whale *Physeter macrocephalus* (Linnaeus 1758) is a globally well‐distributed deep diving toothed whale species that exhibits a strong spatial, sexual, and social segregation between female and young whales in low latitude nursery grounds and adult males at high latitude feeding grounds (Cantor et al., 2019; Eguiguren et al., 2023). The sperm whale population in the Mediterranean Sea is an exception to these long range latitudinal movements (Frantzis et al., 2013; Notarbartolo‐Di‐Sciara, 2014). The species is classified as “vulnerable” under the International Union for Conservation of Nature (IUCN) Red List of Threatened Species (Taylor et al., 2019). The species was previously subjected to extensive commercial whaling from the 18th Century onward, beginning in the North Atlantic and expanding globally as stocks became depleted (Townsend, 1935; Whitehead & Shin, 2022). Whaling ended for the species in the late 20th Century, but the global pre‐whaling population estimate from 1710 of 1.9 million animals had been reduced to an estimated 844,000 individuals in 2022 (Whitehead & Shin, 2022). An estimated abundance of 35,517 individuals (CV = 0.365, 95% CI 17,763–71,016) was determined using data from aerial and shipboard surveys from 2015 to 2017 between the Strait of Gibraltar and Norway (excluding the oceanic archipelagos of Azores, Madeira, and the Canary Islands) (Lacey & Hammond, 2023). Female sperm whales typically remain south of 40–45° N where water temperatures are >15°C around tropical and subtropical nursery grounds where they feed, calve, and reproduce (Best, 1974; Eguiguren et al., 2023; Whitehead, 2018). These grounds occur in the Northeast Atlantic around the volcanic archipelagos of the Azores, Madeira, and Canary Islands between 28 and 39° N (Ferreira et al., 2022; Whitehead, 2018). However, female sperm whales were caught off Galicia in northwest Spain in the late 20th Century at 43° N (Aguilar & Sanpera, 1982). Nursery grounds exist in the western Atlantic around the Caribbean Islands and Gulf of Mexico between North and South America (Gero et al., 2007; Mullin et al., 2021). The female groups found in these grounds comprise of units with a highly social and tightly bonded matrilineal organization structure but are made up of both related and unrelated individuals including immature males (Cantor et al., 2019; Konrad et al., 2018). Group sizes in the North Atlantic can range between 6 and 12 individuals (excluding the presence of calves) (Whitehead et al., 2012). These groups help to defend and raise the groups’ calves, where a huge investment of time and resources are devoted to a calf's development (Cantor et al., 2019).

Male sperm whales leave their female group sometime between 4 and 21 years of age and move to more northern temperate to polar regions in the North Atlantic as they mature, forming “bachelor” groups of sexually mature subadult males (Evans, 1997; IJsseldijk et al., 2018; Whitehead, 2018). Their higher latitude feeding grounds occur in cooler waters off Iceland, Greenland, and Norway as far north as Svalbard at 81° N nearer to the sea ice edge (Bengtsson et al., 2022; Leonard & Øien, 2020; Morange et al., 2023; Pöyhönen et al., 2024; Rødland & Bjørge, 2015; Whitehead, 2018). Males exhibit a northward–southward migratory pattern associated with mating opportunities at the Atlantic nursery grounds (Lefort et al., 2022; Mullin et al., 2021; Steiner et al., 2012).

Off Ireland, the species is found far from shore along the continental shelf in the submarine canyon systems and deep‐sea habitats off the west and northwest coasts of the country (Mackey, 2016). The closest suitable sperm whale habitat to land exists approximately 60 km from shore, but the distance to the shelf edge extends to >380 km, which makes studying this species challenging (Barile et al., 2021; Gordon et al., 2020; Mackey, 2016). The species occurs year‐round in Ireland, as indicated by stranding records, acoustic detections, and sighting records (Berrow et al., 2018; Gordon et al., 2020; Mackey, 2016; Wall et al., 2013).

Sighting surveys have been undertaken in an opportunistic and dedicated capacity in Irish waters from 1980 to the present day (Berrow et al., 2012; O'Brien et al., 2009). While most have been undertaken by ship during petroleum exploration surveys and fisheries assessment surveys, others have been undertaken on dedicated research vessels with the capacity to carry out passive acoustic monitoring through the use of towed hydrophones (Berrow et al., 2012, 2018; O'Brien et al., 2009; Wall et al., 2013). Additionally, fixed wing light aircraft have been used to visually survey for cetaceans, and sperm whales have been detected over the years using a combination of these platforms (Berrow et al., 2012; Rogan et al., 2018).

Acoustic abundance estimates were produced during the ObSERVE Acoustic project for the Irish continental shelf from the Goban Spur along the Porcupine Bank and Rockall Trough regions for 2015 and 2016, which indicated that 3.2 individuals occurred per 1000 km^2^ in habitats deeper than 300 m (Gordon et al., 2020). Sperm whales were detected along the entire continental shelf, but some basin systems displayed higher sperm whale acoustic detection rates (Barile et al., 2021). These basins include the Colm, MacDara, Erris, and Donegal Basins along the continental shelf edge of the Porcupine Bank, West of Ireland (Barile et al., 2021).

Male sperm whales have primarily been recorded in Irish waters due to their location between the nursery grounds around the Azores in the Mid‐Atlantic off Portugal (in addition to other nurseries in Macaronesia) and higher latitude feeding grounds in the North Atlantic extending up into the Arctic Ocean (Evans, 1997). One male sperm whale that was photo‐identified at Andenes, Northern Norway, in 1989 and 1992 was subsequently found stranded in 1997 at Oranmore, Galway, Ireland (Fairley & MacLoughlin, 1997; Steiner et al., 2012). Despite the species’ conspicuous nature, its preferred habitat far from shore is a large factor impairing studies of the species’ life history in Irish waters. Two Irish stranded sperm whales have been necropsied providing an insight into the species diet in detail, one in 2002 comprising of a 14.8 m male and in 2004, a 5.8 m calf (Santos et al., 2002, 2006). Their stomach contents were assessed, revealing a predominantly cephalopod‐based diet of at least 11 squid and octopus identified to species level, in addition to four more at genus level (Santos et al., 2002, 2006). Cephalopods, krill, and fish were noted (not to species level) in the stomach contents of 38 sperm whales examined after being caught by whalers off Northwest Ireland in the early 20th Century (Ryan, 2022). However, little information exists on basic life history areas, such as behavior, habitat use, migratory movements, and population composition. Most detailed information on the species to date has come from strandings and acoustic data (Barile et al., 2021; Gordon et al., 2020).

Climate change is an overarching threat to this species and their habitats globally (Learmonth et al., 2006; Nunny & Simmonds, 2019). The steady warming of the world's oceans, marine heatwaves, ocean productivity, acidification, salinity, storm frequency, reduced sea ice coverage, and ocean circulation alterations that develop have acute effects on habitats and the species that live within them (Nunny & Simmonds, 2019). These impacts are highly dynamic and may affect some habitats and species differently to others who may lose or gain terrain as the climate changes (Welch et al., 2023). These impacts may also affect the individual's welfare and health of individuals through indirect effects of land runoff and the introduction of additional pollutants into the marine environment (Kebke et al., 2022).

A number of toothed and baleen cetacean species have shown distribution shifts northwards in the Northeast Atlantic off Ireland associated with climate change (Evans & Waggitt, 2020; MacLeod, 2009). These include temperate species expanding northwards, such as the common dolphin *Delphinus delphis* (Linnaeus 1758), striped dolphin *Stenella coeruleoalba* (Meyen 1833), Risso's dolphin *Grampus griseus* (Cuvier 1812), goose‐beaked whale *Ziphius cavirostris* (Cuvier 1823), and the dwarf sperm whale *Kogia sima* (Owen 1866), while the cold water favoring species, such as the white‐beaked dolphin *Lagenorhynchus albirostris* (Gray 1846) and Atlantic white‐sided dolphin *Lagenorhynchus acutus* (Gray 1828), have shifted more northwards to remain within cooler waters, but these movements are likely also linked to prey habitat preferences (Chosson et al., 2023; Evans & Waggitt, 2020; Levesque et al., 2023; Plint et al., 2023). Baleen whale strandings (primarily minke whales *Balaenoptera acutorostrata*) along the coasts of Ireland and the UK increased in northern areas between 1990 and 2020, but it was not clear whether climate change was a significant factor in this increase or whether other variables such as increased observer effort or population increase explained the increased prevalence in northern areas compared to the south (Snell et al., 2023).

Sperm whales were a species anticipated to expand their range in response to climate change (MacLeod, 2009). The species was found to be the most sensitive to climate change when its impacts were modeled against the life history characteristics of cetacean species found in the Madeira Archipelago, which was linked to the species diet diversity and low genetic variability (Sousa et al., 2019). Sperm whales have shown a northward expansion as annual sea ice coverage retreats in the Arctic based on both visual observations and acoustic detections as far north as Eclipse Sound, Baffin Bay in Canada to Melville Bay, Greenland in the Northwestern Atlantic at 72–75° N (Frouin‐Mouy et al., 2017; Lefort et al., 2022; Posdaljian et al., 2022). In the Ligurian Sea, Northwest Mediterranean Sea, sperm whale distribution was closely linked with sea surface temperature (SST) (Azzellino et al., 2008). While at Kaikoura in New Zealand, sperm whales were predicted to shift their distribution to higher latitudes in the Pacific Ocean in response to habitat suitability shifts attributed to rising sea surface temperatures (Peters et al., 2022).

In the Bay of Biscay, six sightings of sperm whale calves (totaling seven individuals) were made between the months April and August from 2003 to 2006 (Hobbs et al., 2007). One mother–calf pair was photographed at 44.728, −3.714 in April 2005 (Hobbs et al., 2007). These records represent the closest individuals to the 45° Latitude cut‐off point for the typical female and young sperm whale habitats (Best, 1974; Eguiguren et al., 2023; Whitehead, 2018).

Here, we describe the apparent increasing frequency of female sperm whale strandings in Ireland. Historical and contemporary female, juvenile, and calf sperm whale sightings, strandings, and a whaling catch were reviewed and indicate that Irish waters may have been used to some degree by these animals over the past century but whether they were vagrant individuals or part of a more stable element of the population remains unknown. However, the current trend of female strandings in Ireland indicates that the sperm whale population structure in Irish waters may be altering from a primarily male only structure to include female groups also, which warrants further attention in offshore Irish waters.

## 2 METHODS

Records of sperm whale strandings from the literature and from stranding databases were accessed and reviewed. The Natural History Museum in London's historical cetacean stranding dataset (1913–1989) and the Irish Whale and Dolphin Group's (IWDG) cetacean stranding database (1990–2024) were included in this study (IWDG, 2024; Natural History Museum, 2018). Female whales were confirmed if photographs of the genital area were available, revealing the presence of mammary glands and the lack of a distended penis. When the carcass’ condition of stranded female #10 was degraded due to advanced decomposition, preventing a definitive identification of the individuals sex, a sample was taken for molecular analysis to determine its sex by the author BG and to complement visual characteristics observed by the author SAO'C following Rosel (2003) (process detailed in Supplementary Material S1).

Length measurements were recorded in stranding reports along with photographs of the tail flukes’ trailing edge and the dorsal fin (if suitably intact) for comparison with North Atlantic photo‐identification catalogs to determine if the individual was known. Historical commercial whaling catch data were sourced from the literature (Fairley, 1981; Ryan et al., 2022) and evaluated. Opportunistic sightings of female, juvenile, and calf sperm whales were also searched for at higher latitudes within or near Irish waters (Browne, D., personal communications; Cadhla et al., 2004; ESAS, 2022; O'Brien, J., personal communications; Sigurjónsson et al., 1991; Wall, 2013).

Sperm whale sightings made by experienced marine mammal observers (MMO's) and reported in offshore survey cruise reports were evaluated and compiled together. Additionally, sightings datasets and a sperm whale film made by the general public within and adjacent to Irish waters were also reviewed to determine if any apparent female and/or calf or juvenile individuals had been recorded in the region.

Sea surface temperature (SST) data from Irish offshore waters were accessed from the M1, M3, M4, and M6 weather buoys through the Irish Marine Data Buoy Observation Network (IMDBON) Supplementary Material S2. This network is managed by the Marine Institute in collaboration with Met Éireann, the Irish Meteorological Service and funded by the Department of Agriculture, Food and the Marine. SST was averaged per month for each year of deployment to assess the SST seasonality and spatial spread along the Irish coastal water and the continental shelf to evaluate if suitable conditions exist for female, juvenile, and young sperm whales off Ireland.

## 3 RESULTS

### 3.1 Female records

#### 3.1.1 Whaling

From 1908 to 1914 and again in 1920 and 1922, commercial whaling operations were undertaken off the northwest of Ireland at two land stations operated by Norwegian whaling companies in County (Co.) Mayo (Fairley, 1981). One station was located at Rusheen Island by the South Inishkea Island (54.119, −10.202), while the second station was situated on the mainland at Ardelly Point on the Mullet Peninsula (54.144, −10.060) (Fairley, 1981).

**FIGURE 1:**
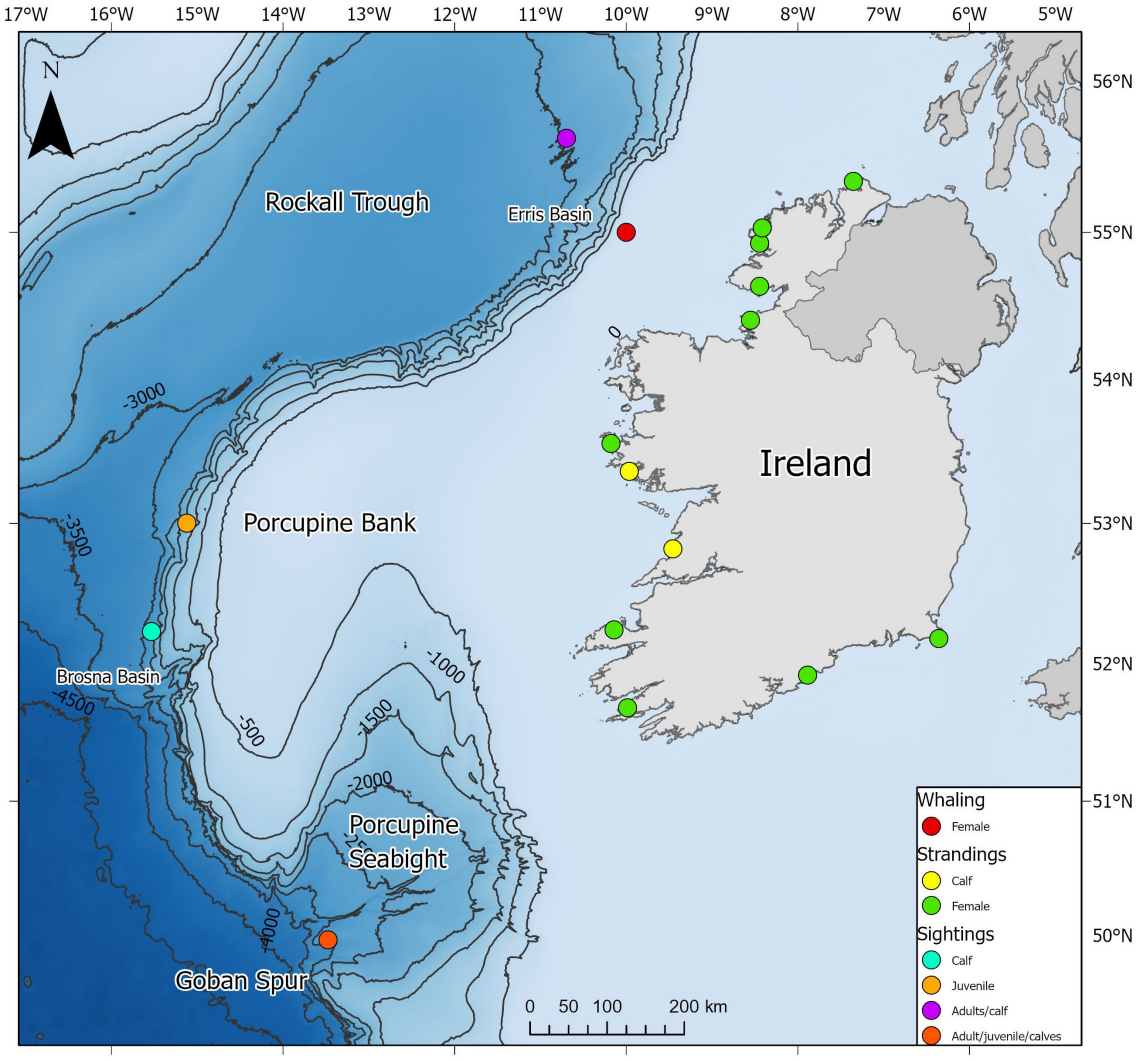
Location of caught, stranded, and sighted adult females, juveniles, and calf sperm whales between 1910 and 2023 off Ireland.

In total, 63 sperm whales were landed at these stations from 1909 to 1914 and in 1920 and 1922 (Fairley, 1981; Ryan, 2022). One female sperm whale was reportedly caught on 18th of June 1910. Its measurement was noted as 14.02 m in length, and its approximate catch location was at 55.000 N, −10.000 W near the Erris Basin and Continental Shelf edge (Figure 1). During the 1910 whaling season, seven sperm whales were caught between both whaling stations from 1 May and 15 September (Fairley, 1981; Ryan, 2022). The other six whales killed that season were males (Fairley, 1981; Ryan, 2022).

#### 3.1.2 Strandings

Strandings of 103 sperm whales have been recorded around the coast of Ireland from 1753 up until 20 April 2024 (IWDG, 2024).

Of these strandings, 51 were confirmed male (49.5%), 42 were unsexed (40.8%), and 10 were female (9.7%). The female whales stranded between 1995 and 2023. The majority of which (*n* = 6) occurred between 2019 and 2023. At least one female stranded each year from 2019 to 2023 (two stranded within one day of each other in February 2022) (Figure 1, Table S1). The female records were primarily concentrated in the northwest of Ireland in Co. Donegal between 54 and 55° N, where four individuals were located between 2001 and 2022 (Figure 1). The breakdown of each stranding case with the information available information available per individual is as follows:

Female #1 live stranded at Redbarn Strand, Youghal, Co. Cork (51.918, −7.884) on 15th of June 1995 (Rogan & Smiddy, 1995). Attempts were made to refloat the whale, but when it died, it was examined that day (Smiddy & Rogan  1995 Smiddy, 1995. Thirteen whale lice sp. were recovered from the animal's dorsal side, and *Penella* sp. were also present (Rogan & Smiddy, 1995).

Female #2 was found at Killybegs, Co. Donegal (54.635, −8.446) on 29th of April 2001. It was a 10 m whale recorded in an advanced state of decomposition (Murphy & Rogan, 2004).

**FIGURE 2:**
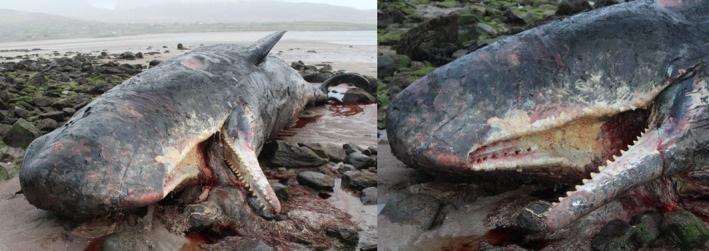
Female sperm whale stranded at Fermoyle, Brandon Bay, Co. Kerry on 22nd of March 2013. Photographs by Seán A. O'Callaghan.

Female #3 stranded dead at Fermoyle, Brandon Bay, Co. Kerry (52.245, −10.141) on the 18th of March 2013. It was a 10.36 m adult whale (Figure 2, O'Connell & Berrow, 2015). The whale was in a moderate state of decomposition and inflated when visited on 20th of March 2013 by SAO'C. Eight maxillary teeth were present in this whale, and one was obtained and donated to the National Museum of Ireland – Natural History's collections reference code NMINH:2018.14.1 (O'Callaghan, 2019). Two mandibular teeth were extracted, and their pulp cavity was occluded, indicating the animal was old with excessive tooth wear (O'Callaghan, 2019).

Female #4 stranded freshly dead at Nethertown, Carnsore Point Co. Wexford (52.183, −6.358) on the 13th of February 2017, it was a 9 m whale (O'Connell & Berrow, 2019).

Female #5 stranded freshly dead at Streedagh Strand, Co. Sligo (54.407, −8.550) on the 3rd of April 2019. It was a 10.3 m individual (O'Connell et al., 2021a). A partial gross necropsy was performed on the beach on 4th of April. The lower mandible had been removed by the general public, but some remaining mandibular teeth were taken. Samples were taken for toxicology (blubber and muscle), in addition to squid eye lenses from a cephalopod sp. by the IWDG (SAO'C, personal observations).

Female #6 stranded dead at Cruit Island, Co. Donegal (55.030, −8.414) on 28th of February 2020. It was a 9.8 m individual (O'Connell et al., 2021b).

Female #7 stranded dead at Claddaghduff, Co. Galway (53.558, −10.178) on 27th of March 2021. It was a 10.2 m whale (IWDG, 2024).

Female #8 stranded dead at Killourt, Malin Head, Co. Donegal (55.340, −7.353) on 15th of February 2022 (IWDG, 2024). It was an 8.5 m whale that displayed an extensive number of sucker mark scars on the head ca. 3 cm in diameter and hemorrhaging around the left side of the head and ear (SAO'C, personal observations).

Female #9 stranded dead at Maghery, Co. Donegal (54.927, −8.444) on 16th of February 2022 (IWDG, 2024). It was an 8.4 m whale in an advanced state of decomposition. Aerial photographs of the carcass confirmed the presence of apparent cookiecutter shark *Isistius brasiliensis* (Quoy & Gaimard, 1824) bite marks present along the entire left lateral flank of the animal. Cephalopod sucker marks were present on the head. Additionally, apparent killer whale *Orcinus orca* (Linnaeus, 1758) rake marks were present on the dorsal fin (5 rakes), on the left pectoral fin (two sets of rakes, one with 5 and the other with 4 rakes), and on the tail stock just posterior to the mammary glands (11 rakes) where more than one raking event appeared to have taken place. The tail fluke was mostly obscured with sand preventing checks for additional rake marks.

Female #10 stranded dead at Eyeries, Co. Cork (51.682, −9.984) in an advanced state of decomposition on 16th of May 2023. It was visited on 20th of May, it measured 6.6 m long, but the tail fluke was not present. The length measurement was done to the last caudal vertebrae present and the true total length was estimated to be over 7 m (SAO'C, personal observations). The whale's skin color was mostly orange, with some remaining skin tissue high on the lateral side of the carcass. No penis was observed, but it was not possible to determine the presence of mammary glands. A blubber sample was used to confirm the whale's sex using a molecular technique described by Rosel (2003), which complemented the observations and measurements of the carcass's overall size and body proportions that were within the expected range for a female whale.

### 3.2 Calf records

A 5.49 m unsexed sperm whale calf was reported stranded at Gorteen Point near Roundstone, Co. Galway (53.366, −9.966) on the 4th of September 1916 (Figure 1, Anon, 1917; Berrow & Rogan, 1997). Eighteen or 19 unerupted teeth were present in its 35 cm long lower mandible that was sawed off and sent to the Museum of Natural History in London (Anon, 1917; Berrow & Rogan, 1997; Harmer, 1917, 1918). No additional details were reported on this individual in the literature, but no subsequent sperm whale strandings were reported from the area at the same time or later that year (Harmer, 1917, 1918).

A 5.8 m male calf live stranded at Spanish Point, Co. Clare (52.819, −9.456) on the 4th of May 2004 after being observed swimming close to the coast in very poor sea conditions (4 m swell and Beaufort sea state 6) (Berrow & O'Brien, 2005). It was observed live stranding twice, but it refloated itself at the time (Berrow & O'Brien, 2005). On the 5th of May, the whale was found stranded dead approximately 2.7 km to the south of the initial stranding location at Quilty Co. Clare (Berrow & O'Brien, 2005).

The animal was necropsied, and the fore stomach was found to contain a small volume of a white liquid identified as milk, as well as 788 upper and 563 lower cephalopod beaks from at least eight species (Begoña Santos et al., 2006). The species that were most abundantly present were the Atlantic cranch squid *Teuthowenia megalops* (Prosch, 1849) (*n* = 73, *N*% = 9.51) and the Elongate jewel squid *Histioteuthis reversa* (A. E. Verrill, 1880) (*n* = 65, *N*% = 8.46) (Begoña Santos et al., 2006).

**FIGURE 3:**
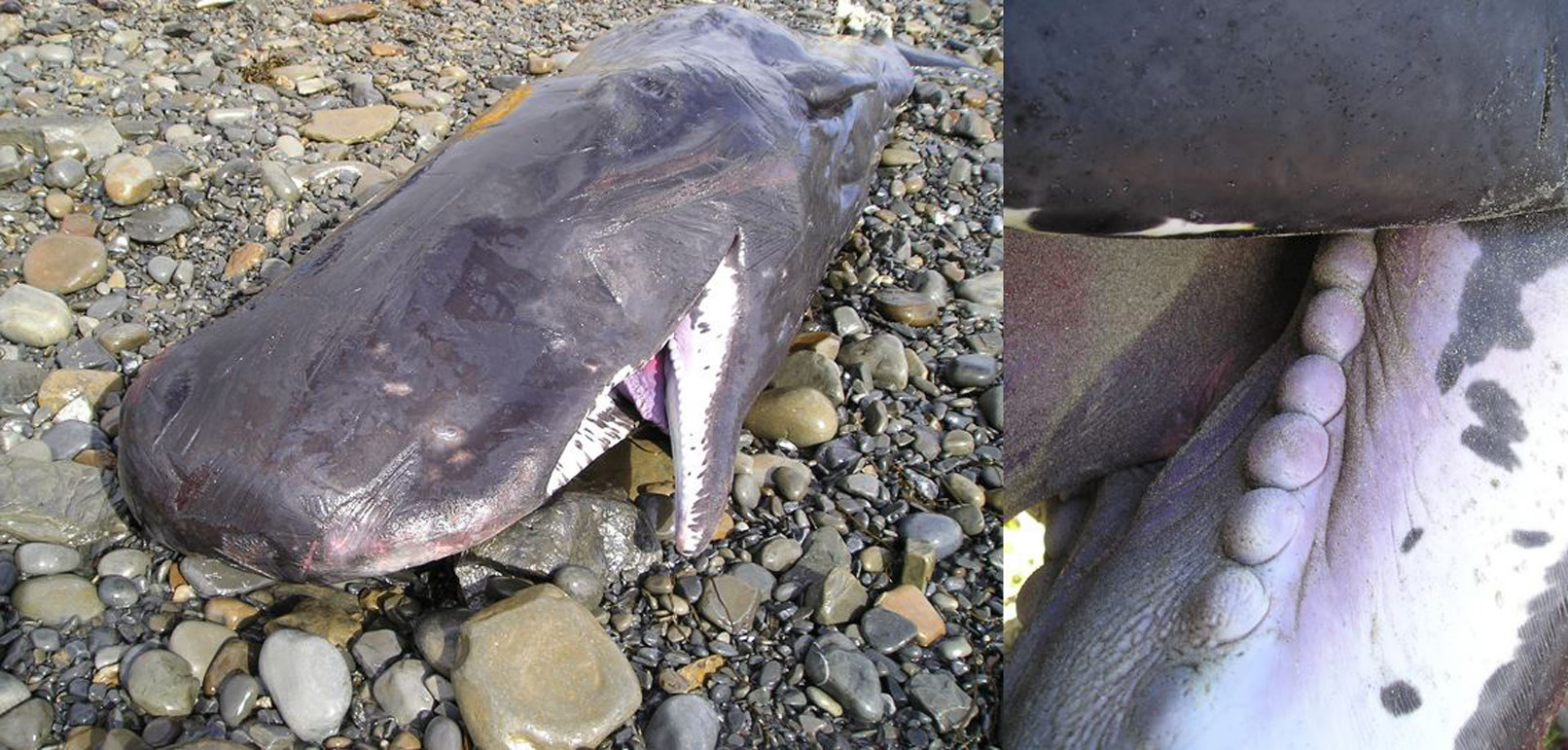
Sperm whale calf stranded dead at Quilty, Co. Clare on 5th of May 2004 displaying protuberances in the lower mandible. Photographs by Dr Simon Berrow.

The lower mandible displayed 20 protuberances when inspected (Figure 3, Berrow & O'Brien, 2005). No associated adult female whale was observed or recorded stranded before or after the calves’ stranding (Philpott et al., 2007).

### 3.3 Sightings

Sperm whales were seen along Ireland's Atlantic Margin during surveys from vessels of opportunity between 1st July of 1999 and 30th of September 2001 (Cadhla et al., 2004). Fifty‐six individuals were observed involving 25 adults, 5 juveniles,3 calves, and 23 unknown age class sperm whales in groups of 1–4 individuals (Ó Cadhla et al., 2004). Three adults and one calf were observed in the Rockall Trough (55.625, −10.694) on 13th of June 2001 (ESAS, 2022; NBDC, 2023). While a sighting at the Goban Spur (49.968, −13.477) occurred on 28th of August 2001 involving four whales, where one was an adult, one juvenile, and two were calves (ESAS, 2022; NBDC, 2023).

It was not possible to account for all sightings of juvenile and calf sperm whale records noted in Ó Cadhla et al., 2004 due to missing records from the reports‐associated database, but two juveniles and the three calf records appear to be accounted for (Figure 1; ESAS, 2022; NBDC, 2023). The data were entered in the European Seabirds at Sea format (ESAS) where the age class attributed was listed as either “Adult” or “Immature,” but more specified classifications were noted by Cadhla et al. (2004) and NBDC (2023) to assist in identifying the whale's age class with the information available.

On 1st of April 2004, a sperm whale calf was observed from RV *Celtic Explorer* during the annual Blue Whiting Acoustic Survey (BWAS) in offshore Irish waters (O'Brien, personal observations).

On 13th of February 2010, a lone juvenile sperm whale was sighted along the shelf edge of the Porcupine Bank during a climate change oceanography survey on RV *Celtic Explorer* (53.0017, −15.1188) (Nolan, 2010; Wall, 2013). The whale was seen alone as it approached the bow of the vessel. There have, however, been no other young sperm whale sightings reported from offshore surveys or studies since this record (Berrow et al., 2018; Rogan et al., 2018).

Additionally, female and calf sightings were reported adjacent to Irish waters during the Icelandic shipboard North Atlantic Sightings Survey (NASS) in August 1989 (Sigurjónsson et al., 1991). A group of nine sperm whales was seen at 52.383, −31.366 approximately 1400 km west of Ireland. Eight of the individuals were estimated to be 10–12 m in length, with one 3–3.5 m long calf which was believed to have recently been born due to its coloration and behavior (Sigurjónsson et al., 1991). The adults swam in close association with one another and performed short 5‐minute dives when observed (Sigurjónsson et al., 1991; Supplementary Material S3).

Another group consisting of 10–12 individuals was observed at 54.066, −28.983 approximately 1300 km off Ireland near the Mid‐Atlantic ridge (Sigurjónsson et al., 1991). Two–four calves were seen within the group (Sigurjónsson et al., 1991; Table S2).

One juvenile was seen with a pair of adults on 3rd of June at the Hatton Bank (58.229, ‐17.629), while a lone juvenile was noted on the 10th of June 2000 also at the Hatton Bank (O Cadhla et al., 2004).

A nursing female sperm whale and young calf were observed and filmed, west of the Mid‐Atlantic Ridge at 46.094, −34.124 from transatlantic rowboat *Cushlamacree* on 7 August 2022 while en route to Galway from New York, USA, for Project EMPOWER (Browne, personal communications; Table S2; Supplementary Material S2).

### 3.4 Sea surface temperature

Averaged monthly sea surface temperature (SST) readings were assessed using readings from data collected by the IMDBON from 2000 to 2023 (Supplementary Material S3 S). Deployment periods and operational times varied depending on the buoys used. The M1 buoy was phased out in 2007, but the other buoys have remained in place and are still recording data (in September 2023) since initially being deployed in 2002 for M3, 2003 for M4, and 2006 for M6 (Marine Institute and Met Éireann, 2023).

Sea surface temperature (SST) values exceeding 15°C were recorded in the months of June – October inclusive across all buoys. The maximum temperature recorded was 17.4°C at the M3 buoy in July 2014. Offshore, the M6 buoy west of the Porcupine Bank displayed a maximum of 16.3°C in September 2007, while the M4 buoy near the Erris Basin northwest of Donegal had a maximum value of 16.4°C in August 2003. The M1 buoy displayed similar >15°C values in each year of deployment except for 2007, when it was recovered on the 25th of March.

When the stranding date of the calf or female sperm whales was compared with monthly SST, the SST varied considerably from 9.85 to 16.69°C during the month when strandings were recorded while the IMDBON was active (2000–2023) (Table S3).

Sighted sperm whale calves and juveniles in Irish waters were seen along the Porcupine Bank, where the nearest weather buoy to the sightings was the M6 buoy. It was not in operation when the calf was observed in 2004, so the nearest buoy at the time was the M1, which recorded an SST of 10.40°C in April of that year. The 2010 observation of a juvenile coincided with a measurement of 11.15°C from the M6 buoy (Table S4).

## 4 DISCUSSION

The presence of female, juvenile, and calf sperm whales at higher latitudes than within the range of the nursery grounds in the Northeast Atlantic has been identified off the coast of Ireland using the available historical and contemporary data from whaling, stranding, and sighting records between 1910 and 2023.

It appears that the population structure for sperm whales off Ireland began to change over the past decade from a primarily male‐based population to now include females, as indicated by these stranding and sighting records. The presence of a female and calf in the early 1900s may indicate the presence of vagrant whales a century ago but the sighting records since 1989 and increase in frequency in strandings from 2019 onwards indicate that female sperm whales have become more frequent off Ireland.

Length estimates were made using the interpulse interval (IPI) from sperm whale acoustic recordings in Irish waters using both passive (towed hydrophone) and static acoustic recorders between 2014 and 2016 (Barile et al., 2024). Acoustic data were collected from the Goban Spur and Porcupine Seabight between 49.547, −13.373 and 51.381, −11.624 north to the Erris Basin of the Porcupine Bank at 55.630, −9.730 (Barile et al., 2021, 2024). The majority of the estimated whales were within the size range of adult females or juvenile males (between 9 and 12 m) at 49% (*n* = 788), while immature individuals (<9 m) accounted for 25% (*n* = 394) of the total individuals with acoustic length estimates (*n* = 1595) (Barile et al., 2024). There was an indication of size segregation with smaller whales recorded in the southern section of Irish waters (Barile et al., 2024). Acoustic length estimation methods are somewhat constrained, given that if whales <7.75 m were present, they would not be detected using the currently available IPI length estimation equations (Barile et al., 2024).

This population shift brings with it different conservation management considerations, given female groups tend to show a higher degree of site fidelity, may possess multiple individuals and, most crucially, contain calves. Such groups may be more susceptible to anthropogenic disturbance (or at least can be affected in an alternate manner) than males who may forage widely or just transit through Irish waters while on migration (Steiner et al., 2012). Offshore Irish waters are exposed to acoustic anthropogenic sound sources, such as seismic surveys for oil and gas, development of ocean renewable energy sources, and an apparent increase in military activities involving foreign navies in recent years, which may pose a risk to groups of female, juvenile calf sperm whales, in addition to other deep diving cetacean species (Beck et al., 2013; Kavanagh et al., 2019; Miller et al., 2022; O'Callaghan et al., 2022; Thompson et al., 2020).

Female sperm whales reportedly reach sexual maturity at approximately 9 years of age and 9 m in length, and physical maturity occurs when growth ceases at around 30 years old at 10.6 m in length (Whitehead, 2018). The gestation period of sperm whales is estimated to be between 14 and 16 months and pregnancy occurs at 5‐year intervals (Best, 1979; Clarke, 1956). When born, calves are between 3.7 and 4.1 m long, averaging 3.93 m in the Northeast Atlantic (Clarke, 1956). Nursing can last from 13 to 24 months (Best, 1979; Clarke, 1956).

The two calf stranding records from Ireland in 1916 and in 2004 indicate that at least one adult female was likely in the vicinity of the stranding, given they were still at a size where the calf would depend on the female for nursing (5.49 and 5.8 m), especially given the presence of likely milk (white liquid) in the stomach of the 2004 animal (Santos et al., 2006). It is most likely that these calf cases involved more than one adult female, given the social group behavior exhibited by the species, and the calf sighted one month previous to the 2004 stranding along the Porcupine Bank may have been waiting at the surface for the accompanying adult or its group to resurface after foraging when it was observed (Cantor et al., 2019). However, it was not piossible to confirm the presence of any other associated whales.

Six of the Irish female stranding records exceeded 9 m indicating they were sexually mature, while the 1910 whaling record was reportedly 14.02 m, which indicates that these animals were mature individuals. The 14.02 m individual may be an extremely large individual, or there may have been some inaccuracy in its total length measurement (Best, 1989).There was also the potential for the animal to be mistaken for a female if the penis was not distended due to decomposition gases after death as noted on occasion in the Azores (Clarke 1956). The other sperm whales caught in the 1910 season were confirmed to be males and the distance required to tow a whale back to the land station typically resulted in such carcasses to degrade by the time they were landed, a number were noted to have exploded when the pressure was released while biologists examined the animals on the flensing plane so it should have been obvious that the whale was a male at that point (Fairley 1981). These results indicated that the majority of female sperm whale stranding records from Irish waters since 1995 were of sexually mature individuals (*n* = 6).

Sperm whale strandings in the North Sea (involving male or unsexed individuals) showed a link with positive temperature abnormalities and may be linked to their cephalopod prey's preference for such oceanographic conditions (Pierce et al., 2007). Heightened SST conditions during El Niño southern oscillation events were found to be a factor in lowered conception rates for female sperm whales at the Galápagos Islands for about 1.5 years after such events took place when foraging success was suppressed by warmer water conditions (Whitehead, 1997).

In the western Atlantic, female and immature sperm whales have been recorded as far north as “the Gully” off Nova Scotia, Canada, where a presumed group of females was observed at approximately 44.000, −59.000 on 23rd June 1990 (Whitehead et al., 1991). Female groups with juveniles and calves were also observed on occasion in the New York Bight, USA, between 2018 and 2019 (Zoidis et al., 2023). A sighting record of a sperm whale mother–calf pair also occurred at 43.440, −36.850 west of the Mid‐Atlantic Ridge off of Newfoundland, Canada, in June and July 2018 (Wachtendonk et al., 2023). A group of eight juvenile‐sized sperm whales were also observed during this 2018 Canadian survey (Wachtendonk et al., 2023). Acoustic length estimates were produced for sperm whales south of Martha's Vineyard and Nantucket, Massachusetts, USA, from 40 to 41° N using the interclick interval (ICI) between 2020 and 2022 (Westell et al., 2024). “Social groups” of female and immature‐sized whales (8–11 m) were recorded in their region with immature and mature male‐sized individuals (Westell et al., 2024).

Additionally, in the Pacific, female sperm whales and immature individuals have been observed off the Aleutian Islands, Alaska, USA at 52.000, −174.000 where historically there were some whaling records of females caught north of 50° N in both the Gulf of Alaska and Kamchatka, Russia (Fearnbach et al., 2012; Whitehead, 2018). These observations are supported by IPI length estimates for both the Gulf of Alaska and the Bering Sea/Aleutian Islands from between 2010 and 2019 from 52 to 58° N (Posdaljian et al., 2024 ). IPI length estimates were produced for 3047 animals where 2387 displayed a median length of 10.2 m which was within the size range for female whales (Posdaljian et al., 2024).

There was no major historical precedent for northern female or calf sperm whale records in the Northeast Atlantic apart from a few commercial whaling catches in Scotland, which may have been vagrant females, given the nearest regular catch location for females in the 20th Century was off Galicia, Northwest Spain (Aguilar & Sanpera, 1982; Ryan et al., 2022).

Sperm whales were subject to pelagic whaling in the North Atlantic beginning in 1761 from the east coast of the USA when the Yankee whaling fleet began targeting the species (Townsend, 1935). These sail boats primarily operated in tropical and subtropical whaling grounds between 28 and 33° N in the North Atlantic. However, the furthest north whaling grounds called the “Commodore Moore Ground” were located between 47 and 51° N, 20–25° W ranging from 880 to 1000 km to the southwest and west of Ireland (Townsend, 1935). These grounds were used during the summer months and were noted as having moderate sea temperatures that were influenced by the North Atlantic Drift of the Gulf Stream (Townsend, 1935). The southern extent to which these whaling ships operated undoubtedly biased the latitudinal spread of their catches and omitted more northern areas where female sperm whales may have been present historically to some degree but went underrecorded, given 51° N was the furthest north sperm whaling vessels operated to (Townsend, 1935).

Sperm whales have previously been recorded being predated on by killer whales elsewhere in the world (Gemmell et al., 2015; Pitman et al., 2001). But in the Atlantic, limited reports have been published on the species interacting with one another. In the eastern Atlantic, harassment has been noted toward sperm whales from killer whales off Angola, Western Africa where a group of 18–20 sperms were harassed by five killer whales and blood‐filled spray was observed on two occasions from a sperm whale (Weir et al., 2010). A neonate sperm whale calf with its umbilicus present was found freshly dead with extensive bruising and tooth rake marks consistent with killer whale rakes in Gabon (Weir et al., 2010). In the western Atlantic, defensive behavior was exhibited by a group of female sperm whales off the Bahamas and harassment by killer whales toward sperm whales in the Gulf of Mexico and off the coast of Brazil (Dunn & Claridge, 2013; Sucunza et al., 2022; Whitt et al., 2015). Offshore killer whales are known to eat fish off Ireland (O'Brien et al., 2009; Ryan & Holmes, 2012), and the last two remaining male killer whales from the West Coast Community that frequents Irish and UK waters are known to predate on small marine mammals (Beck et al. 2014). It is likely that the rake marked individual stranded at Maghery, Co. Donegal on the 16th of February 2022 encountered whale hunting killer whales at more southern latitudes.

Marine heatwaves have increasingly become a concerning issue for the world's oceans, given that they permeate into deepwater habitats and may affect depths down to 200 m, while the duration of their effects also increases with depth (Fragkopoulou et al., 2023). Sea surface temperatures increased during a warm period from the mid‐1990s to mid 2000s peaking in 2007 when Irish waters were on average 0.8°C warmer than from 1960 to 1990 (McCarthy et al., 2023). However, Irish waters have since cooled at a rate of −0.3°C/decade since then but still remain 0.4°C warmer in the 21st Century when compared to 1960–1990; this cooling was linked to a weakening of the Atlantic Meridional Overturning Circulation (AMOC) (McCarthy et al., 2023). Additionally, the influence of the Atlantic Niño/Niña events likely plays a role in the dynamics of predator and prey distributions as Niño impacts on SST value variability in tropical waters centered around the Equator and also to the north of this region (Chikamoto et al., 2020; Kim et al., 2023). So, it remains to be seen how offshore Irish waters alter as the effects of climate change become clearer in the years ahead, but the apparent increasing presence of female sperm whales is a signal of widespread habitat change for the species and other top predator communities.

The lack of a correlation between the recorded SST values and the confirmed stranding records of female and calf sperm whales from 2001 to 2023 suggests that SST should not be considered as the sole factor limiting female whales to lower latitudes. SST was recorded at 9.2°C on 23rd June 1990 when the presumed group of female sperm whales was observed in the Gully off Newfoundland, which further suggests that SST may not be a significant factor in the presumed limited range of female sperm whales in the North Atlantic (Whitehead et al., 1991). Prey distribution shifts from historically favorable habitat into new grounds are likely a large factor in the apparent increasing presence of female sperm whales off Ireland.

However, the extent to which they occur, and the dynamics of Ireland's sperm whale population, are currently understudied, and more research resources are required to study sperm whales and other elusive offshore cetacean species from within Irish offshore waters. Sperm whales face a variety of conservation issues, and knowledge of the population's structure and dynamics in the open ocean is required to potentially tailor meaningful conservation management actions to safeguard the species.

## AUTHOR CONTRIBUTIONS

**Seán A. O'Callaghan:** Conceptualization (lead); data curation (lead); formal analysis (lead); funding acquisition (lead); investigation (lead); methodology (lead); resources (lead); software (lead); validation (lead); visualization (lead); writing – original draft (lead). **Bogna Griffin:** Formal analysis (supporting); investigation (supporting); methodology (supporting); resources (supporting); validation (supporting); writing – review and editing (equal). **Stephanie Levesque:** Data curation (equal); investigation (supporting); methodology (supporting); resources (equal); validation (supporting); writing – review and editing (equal). **Martin Gammell:** Investigation (supporting); methodology (supporting); resources (supporting); supervision (supporting); validation (supporting); writing – review and editing (equal). **Joanne O'Brien:** Conceptualization (supporting); data curation (supporting); formal analysis (supporting); investigation (supporting); methodology (supporting); project administration (lead); resources (supporting); supervision (lead); validation (supporting); visualization (supporting); writing – review and editing (equal).

## FUNDING INFORMATION

This study received funding from the National Parks and Wildlife Service (NPWS) small recorders grant 2023 (SPU RG92‐2023).

## CONFLICT OF INTEREST STATEMENT

The authors declare no competing interests.

## Supporting information


Data S1:

## Data Availability

All data are included in the manuscript under the Tables S1–S4.
