# Supplementary material for: Female, juvenile, and calf sperm whale Physeter macrocephalus (Linnaeus 1758) records from Ireland
Source: Ecol Evol. 2024 Sep 1;14(9):e70056. doi: 10.1002/ece3.70056 (PMC11366682; doi:10.1002/ece3.70056)
Supplement: Supplementary file 1 — Data S1: [file ECE3-14-e70056-s001.docx]

**Supplementary Material 1**

DNA from ethanol DNA was extracted from the skin, blubber and muscle using automated Qiacube connect (Qiagen) and the QIAamp DNA mini extraction kit (Qiagen) following the manufacturer's protocol. The extracted DNA was pooled, and a multiplex PCR reaction as per Rosel 2003 with slight modifications was run with the following primer pairs SRY Gene: TtSRYR ACCGGCTTTCCATTCGTGAACG PMSRYF CATTGTGTGGTCTCGTGATC (339bp amplicon size) and ZFX Gene: ZFX0582F ATAGGTCTGCAGACTCTTCTA, ZFX0923R AGAATATGGCGACTTAGAACG (382bp amplicon size) both neat and 1 in 10 dilution. The PCR set up was: 12.5µl of Qiagen Multiplex PCR kit Master mix (2X), 0.06µM of TtSRYR primer and 0.03µM of PMSRYF, ZFX0582F, ZFX0923R. All primers had a starting concentration of 10µM. 5µl of Q-solution and 3.58µl bovine serum albumin (BSA) were also added for a total reaction volume of 25µl which included 2µl of template DNA. Negative template controls (NTC), negative extraction controls (NPC) and positive controls for males and females were included in the PCR test.

Reactions were run on a PCR Max Alpha Cycler (PCRmax, Staffordshire, United Kingdom) under the following conditions: an activation step at 95°C for 5 min, followed by 35 cycles of denaturation at 95°C for 30 sec, annealing for 90 sec at 51°C, extension for 30 sec at 72°C, and a final elongation step for 10 min at 72°C.

PCR products were examined by agarose gel electrophoresis (3% w/v) in a 1X Tris/Borate/EDTA buffer solution with Ethidium Bromide fluorescent dye (EtBr), Molecular Grade (Promega) (12µl in 100µl gel) and the use of BIO-RAD Molecular Imager GelDocTM XR System with the associated Quantity One ®1D Analysis Software version 4.6.1.

**Supplementary material 2**


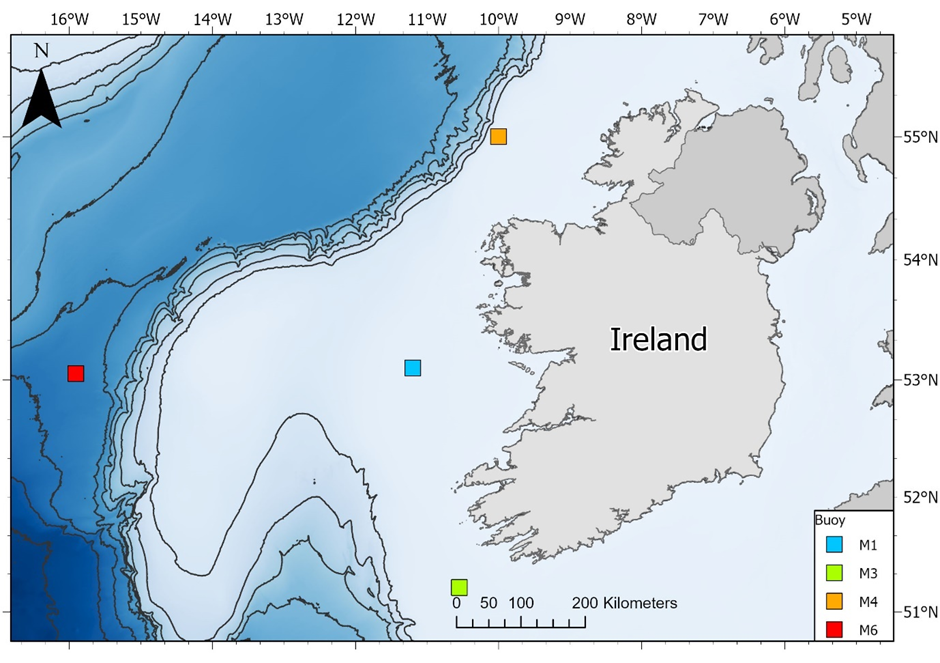


**Irish Marine Data Buoy Observation Network (IMDBON) buoys used during this study for monthly sea surface temperature readings from 2000 – 2023.**

**Supplementary material 3**


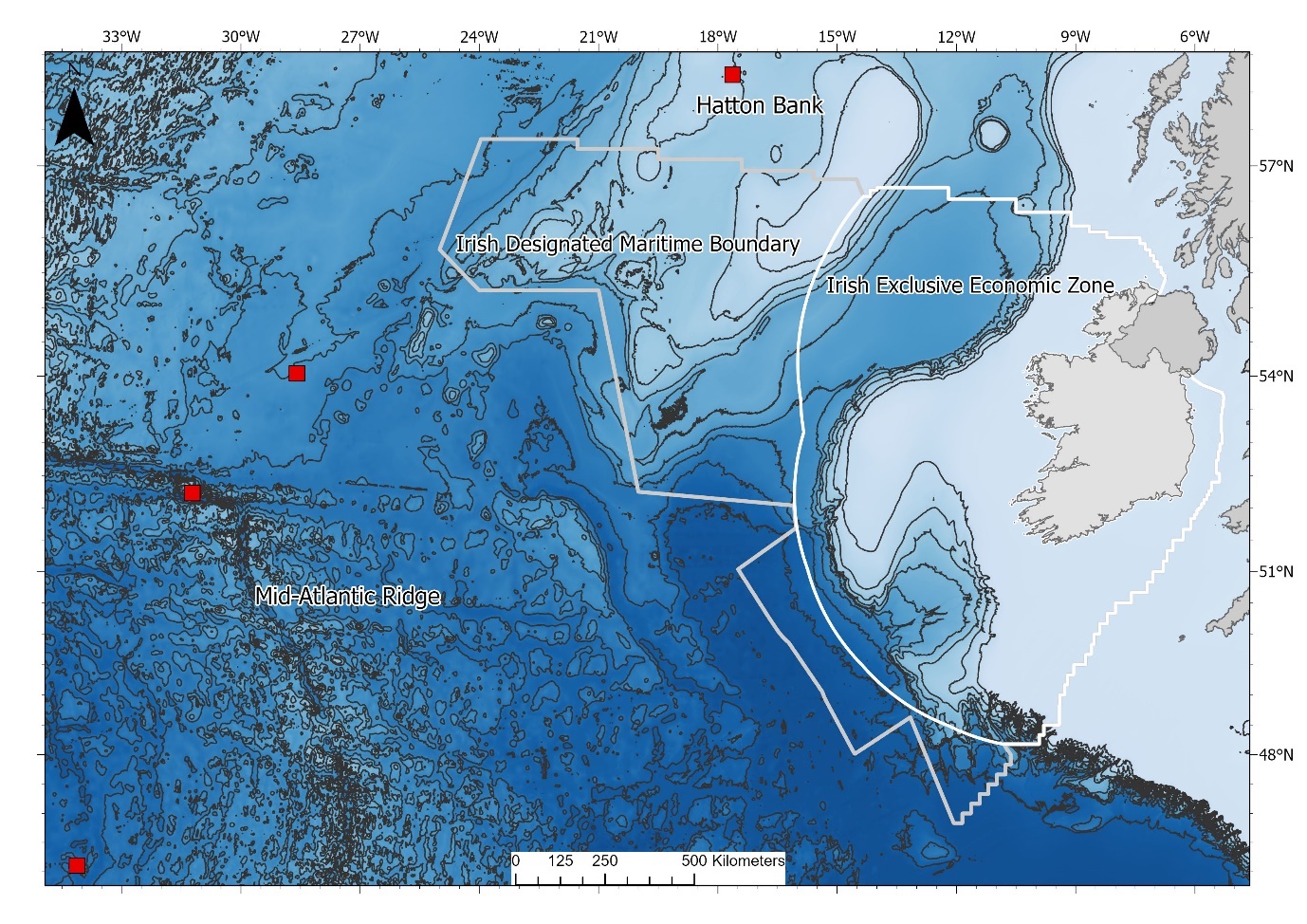


**Location of female sperm whales and calves adjacent to Irish waters in 1989, 2000 and 2022.**

**Table 1. Records of adult female, juvenile and calf sperm whales killed, stranded and sighted from Ireland 1910 - 2023**

~length from head to first exposed caudal vertebrae because the fluke had detached from the body.

**Table 2. Sperm whale juvenile and calf records adjacent to Irish waters 1989 - 2022**

**Table 3. Female sperm whale and one calf stranding 2001 – 2023 in relation to recorded SST (°C) values in Irish waters by the Irish Marine Data Buoy Observation Network (IMDBON)**

When data was not available for the specific stranding month, the nearest available month was selected (~Jan, *Feb, +Mar, ^April, #June)

**Table 4. Sperm whale calf and juvenile sightings along the Irish continental shelf edge in relation to recorded SST (°C) values in Irish waters by the Irish Marine Data Buoy Observation Network (IMDBON)**
